# Reliability of gaze-contingent perimetry

**DOI:** 10.3758/s13428-023-02225-y

**Published:** 2023-09-11

**Authors:** Nikita Thomas, Jennifer H. Acton, Jonathan T. Erichsen, Tony Redmond, Matt J. Dunn

**Affiliations:** 1 School of Optometry and Vision Sciences, Cardiff University, Maindy Road, Cardiff, Wales, CF24 4HQ UK; 2 Vision and Hearing Sciences Research Centre, Anglia Ruskin University, East Road, Cambridge, UK

**Keywords:** Perimetry, Eye tracking, Visual fields, Gaze-contingent, Nystagmus

## Abstract

Standard automated perimetry, a psychophysical task performed routinely in eyecare clinics, requires observers to maintain fixation for several minutes at a time in order to measure visual field sensitivity. Detection of visual field damage is confounded by eye movements, making the technique unreliable in poorly attentive individuals and those with pathologically unstable fixation, such as nystagmus. Microperimetry, which utilizes ‘partial gaze-contingency’ (PGC), aims to counteract eye movements but only corrects for gaze position errors prior to each stimulus onset. Here, we present a novel method of visual field examination in which stimulus position is updated *during* presentation, which we refer to as ‘continuous gaze-contingency’ (CGC). In the first part of this study, we present three case examples that demonstrate the ability of CGC to measure the edges of the physiological blind spot in infantile nystagmus with greater accuracy than PGC and standard ‘no gaze-contingency’ (NoGC), as initial proof-of-concept for the utility of the paradigm in measurements of absolute scotomas in these individuals. The second part of this study focused on healthy observers, in which we demonstrate that CGC has the lowest stimulus positional error (gaze-contingent precision: CGC = ± 0.29°, PGC = ± 0.54°, NoGC = ± 0.81°). CGC test–retest variability was shown to be at least as good as both PGC and NoGC. Overall, CGC is supported as a reliable method of visual field examination in healthy observers. Preliminary findings demonstrate the spatially accurate estimation of visual field thresholds related to retinal structure using CGC in individuals with infantile nystagmus.

**Supplementary Information:**

The online version contains supplementary material available at 10.3758/s13428-023-02225-y.

## Introduction

Standard Automated Perimetry (SAP) is used to examine the integrity of the visual field and is the current gold-standard for detecting and monitoring visual field loss in glaucoma. It is well known that the technique is limited in its ability to identify early damage (Keltner et al., 2000; Tafreshi et al., 2009) and that variability increases with advancing visual field damage (Artes et al., 2002; Gardiner et al., 2014). Glaucoma frequently presents alongside other co-morbidities in patients attending eye clinics. Yet, published reports on the utility and performance of SAP have, for the most part, been evaluated in patients presenting with glaucoma only. When developing and evaluating perimetric techniques, the deleterious effects of the eye’s optical system (including blur and intraocular stray light) are often considered as it is relatively straightforward to disentangle these effects from the effects of neural loss in glaucoma. However, the impact of conditions affecting fixation stability is often overlooked, posing challenges when measuring sensitivity and evaluating the performance of SAP in these patients. It is important for clinicians to measure the visual field in patients who have such conditions and are also at risk of glaucoma or its progression.

One co-morbidity that makes perimetry particularly difficult to perform is infantile nystagmus; a lifelong condition characterized by constant involuntary eye movements and reduced vision. Notwithstanding the difficulties of measuring the visual field in patients with nystagmus, these patients are still at risk of developing glaucoma, and as such, require an accurate measure of the visual field in order to investigate whether or not they have glaucoma or any progression of established damage. It is likely that eye movements increase perimetric variability as the image of the stimulus is smeared across the retinal ganglion cell density gradient, making it even more difficult to measure true visual field loss in these individuals. People with infantile nystagmus do not usually experience oscillopsia (oscillation of the visual scene) despite the presence of continuous eye movements. This results in a disconnect between the perceived visual field and retina. If the goal of SAP were solely to assess integrity of the *perceived* visual field, then involuntary eye movements would be inconsequential. However, in clinical investigations of conditions such as glaucoma, SAP sensitivity is sometimes combined with (or considered in conjunction with) measures of retinal structure in the identification of glaucomatous changes. Consequently, the effect of eye movements is still an important consideration, especially when investigating highly localized relationships between structure and function.

Even when fixation is perceived by patients with nystagmus to be stable, a stimulus of 200 ms duration undergoes a steady change in sampling as its image is moved across the visual field (e.g., a change in retinal ganglion cell density, level of neural convergence along the visual pathway, and local spatial summation) which, in those with steady fixation, would ordinarily contribute to the visual field sensitivity measurement at any fixed location. With such changes in stimulus sampling, it stands to reason that variance around the estimate of visual field sensitivity, as well as variance in nystagmus cohort ‘norms’, are likely to be greater than in normally sighted non-nystagmat individuals.

**Fig. 1:**
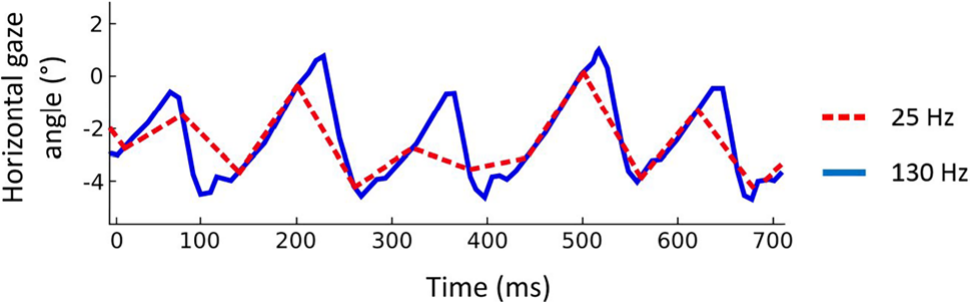
Example of an infantile nystagmus waveform tracked at 130 Hz (*blue line*) and the same waveform downsampled to 25 Hz (*red dashed line*). Maximum error between sampling rates: 2.31° at 359 ms

It is essential that individuals with nystagmus have equality of access to eye care, which includes routine clinical tests such as perimetry to aid the detection of pathology. Although the visual field is perceived to be stable in infantile nystagmus (Bedell & Bollenbacher, 1996; Bedell & Currie, 1993; Goldstein et al., 1992; Leigh et al., 1988), neither the prevalence nor the visual consequences of common abnormalities in the visual field (e.g., glaucoma) are well understood in these individuals. Combining visual field assessment and high-resolution eye tracking allows for the presentation of gaze-contingent stimuli and provides a method to reduce the changes in stimulus sampling with eye movements in infantile nystagmus. One method, known as microperimetry (sometimes referred to as ‘fundus-automated perimetry’), aims to incorporate tracking of the retinal image to enable gaze-contingent stimulus presentation (Crossland et al., 2012). However, most commercial microperimeters are only *partially* gaze-contingent (PGC): the stimulus is initially presented at the appropriate retinal location, but its position is not updated to compensate for eye movements made *during* stimulus presentation. Therefore, any eye movements that occur *during* a stimulus presentation will ‘smear’ the image of the stimulus across the retina. One recent microperimeter, the Compass Fundus Automated Perimeter (CenterVue, Padova, Italy), does allow for compensation of eye movements during stimulus presentation but the rate of fundus tracking is limited to 25 Hz (Rossetti et al., 2015). While 25 Hz might be a sufficient sampling rate for identifying significant changes in the preferred retinal locus, Jones et al. (2016) showed that it is too slow to compensate for quick changes in fixation caused by saccades and microsaccades during visual field examination in children with stable fixation. This sampling rate is also too slow for precise tracking of eye position in patients with infantile nystagmus (see Fig. 1).

The aim of the present study was to investigate the ability of complete, or ‘continuous’ gaze-contingency (CGC) to measure the edges of the physiological blind spot in infantile nystagmus compared to PGC and the standard ‘no gaze-contingency' (NoGC) method of visual field examination. Additionally, we investigated the ability of gaze-contingent stimuli to continually stimulate intended areas of the visual field in healthy observers. This was determined for each test paradigm (CGC and PGC) by comparing measures of the ‘stimulus positional error’ with those from the standard NoGC test (control). This error reflects the gaze-contingent stimulus update delay related to the monitor refresh rate (for detail see ‘Statistical analysis’). Furthermore, this study investigated the accuracy and precision of visual field sensitivity estimates in healthy participants with CGC as a baseline for additional future measurements in infantile nystagmus. Accuracy was determined for each test paradigm by comparison of sensitivity measures with those from standard NoGC (see ‘Supplementary materials’). Precision was determined by the variability in measurements between repeat tests conducted within a short timescale (test–retest variability), as well as by investigating the presence of any learning effects (see ‘Supplementary materials’). The relative contributions and utility of gaze-contingency and stimulus duration were determined from these comparisons.

## Methods

This study consisted of two parts: (1) case examples demonstrating the ability of CGC to detect an absolute physiological scotoma in individuals with infantile nystagmus, and (2) a cross-sectional study of healthy observers comparing the accuracy and precision of visual field sensitivities between CGC, PGC, and NoGC methods of visual field examination. Each method of visual field examination was conducted with the following paradigms developed by the authors:

### Continuous gaze-contingency (CGC)

Visual field sensitivity was measured while continuously updating stimulus presentation location based on eye tracking at a sampling rate of 2000 Hz. The continual update of stimulus position with CGC causes the stimulus to *move* according to the latest available gaze position for the entire duration of the presentation, thus keeping the image of the stimulus as close to the same retinal locus as possible.

### Partial gaze-contingency (PGC)

Visual field sensitivity was measured with the stimulus position initially presented relative to the last known gaze position (also based on eye tracking at a sampling rate of 2000 Hz), but not updated in response to eye movements made *during* stimulus presentation.

### No gaze-contingency (NoGC)

Visual field sensitivity was measured with stimuli presented in their original locations, with no compensation for eye movements.

### Participants

For the first part of this study, three participants with infantile nystagmus were recruited from the Cardiff University Research Unit for Nystagmus cohort and three age-matched controls were recruited from the School of Optometry and Vision Sciences at Cardiff University. Participants with infantile nystagmus with any history of eye disease (other than infantile nystagmus and congenital conditions associated with infantile nystagmus) or ocular trauma were excluded. For age-matched controls, the inclusion criteria were corrected visual acuity of 0.00 LogMAR or better, mean refractive error of ≤ ± 6.00 diopter sphere in any meridian in the test eye, intraocular pressure ≤ 24 mmHg as measured with a non-contact tonometer (Pulsair Desktop Tonometer, Keeler Ophthalmic Instruments, Windsor, UK), and lenticular opacities no greater than grade two in any category of the Lens Opacity Classification System (LOCS III) (Chylack et al., 1993). All age-matched controls underwent a standard ophthalmic examination including visual field examination with the SITA Standard 24–2 program on a Humphrey Field Analyzer (HFA) III (Carl Zeiss Meditec, Dublin, CA, USA) for confirmation of normal visual field status (no clusters of three or more PD abnormalities at the *p* < 5% level in any region of the visual field [Hodapp–Parrish–Anderson grading scale for glaucomatous damage; (Hodapp et al., 1993)] and ‘within normal limits’ on the Glaucoma Hemifield Test). An optometrist conducted fundoscopy to confirm normal optic nerve head and fundus appearance of age-matched control participants. Furthermore, age-matched control participants with a history of any visual system disorder (e.g., glaucoma, diabetes, ocular hypertension), ocular trauma, and a family history of glaucoma were excluded. In both groups, participants taking medications known to affect visual function were excluded.

For the second part of this study, 33 healthy observers (median [IQR] age: 25 years [23, 26.8]; 18 females) were recruited from staff and students at the School of Optometry and Vision Sciences at Cardiff University. Inclusion criteria for these participants were identical to the criteria for age-matched controls in the first part of this study.

### Apparatus

For both parts of this study, stimuli were presented on a gamma-corrected 21″ cathode-ray tube display (Sony Trinitron CPD-G520, with a luminance range of 0.049–84.64 cdm^-2^, resolution 1280 × 768 pixels, refresh rate 130 Hz), via a Bits# stimulus processor (Cambridge Research Systems, Rochester, UK) run in ‘Mono++’ mode (14-bit depth), in an otherwise dark room. A shroud of black card was used to eliminate stray light. The Bits# stimulus processor enabled stimuli to be presented to the nearest 0.001 cdm^-2^. Horizontal and vertical gaze position were recorded in screen-based (pixel) gaze coordinates with an EyeLink 1000 Plus (a video-based eye tracker; SR Research, Ontario, Canada; firmware version 5.12) at 2000 Hz using a Tower Mount. For participants with infantile nystagmus, the eye tracker was calibrated using the method described by Dunn et al. (2019). For healthy observers with stable fixation, eye tracker calibration was performed using the built-in (five-point) EyeLink calibration procedure. For both participant groups, a single-point drift correction (calibration matrix translation) was performed after every 100 stimulus presentations. The head was stabilized by a chin and forehead rest. Stimuli were generated with experiment code written in MATLAB (version 2018a, The MathWorks, Inc., Natick, MA, USA), using the Psychophysics Toolbox extensions (Brainard, 1997; Kleiner et al., 2007; Pelli, 1997).

### Stimulus duration

**Fig. 2:**
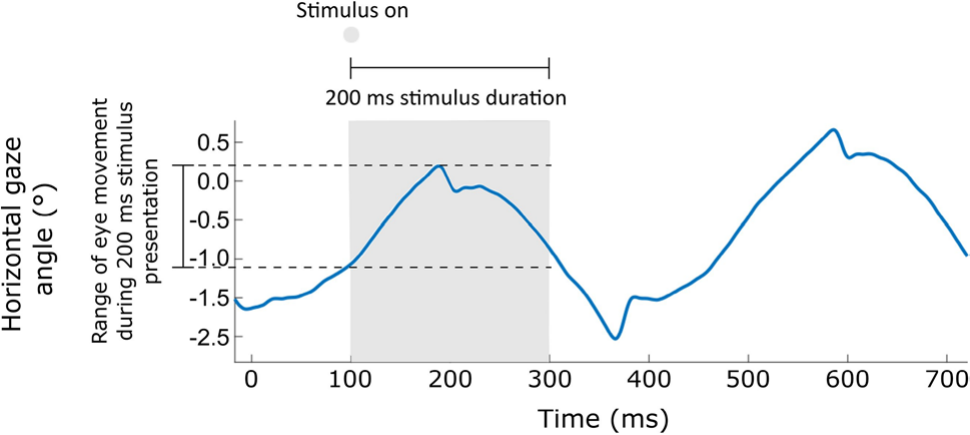
Example of an infantile nystagmus waveform, demonstrating the extent of eye movement that can occur during a 200 ms stimulus presentation. In this example, there has been approximately 1–2° of eye movement during presentation of the stimulus, which would lead to a PGC or NoGC stimulus ‘smearing’ across the retina

An alternative method to reduce changes in the sampling of the stimulus during presentation in individuals with infantile nystagmus is to reduce stimulus duration. Durations within the range of 100–200 ms were first adopted in conventional perimetry to avoid the effects of temporal summation (Aulhorn & Harms, 1972) and to avoid stimuli being missed during blinks (International Council of Ophthalmology, 1979). However, the longer the stimulus duration, the greater the effect of stimulus image ‘smear’ across the retina (Fig. 2).

The critical duration in healthy observers has recently been reported as approximately 30 ms for stimuli commonly used in perimetric examinations, with only partial summation occurring for durations in the range of 30 to 198.3 ms (Mulholland et al., 2015a). Moreover, the critical duration is increased in glaucoma, meaning that the range of stimulus durations for which total luminous energy remains constant at threshold is also enlarged (Mulholland et al., 2015b).

Both parts of this study conducted CGC, PGC, and NoGC examinations using two distinct durations: 30 and 200 ms (in separate examinations). These durations were chosen as a reference to the approximate critical duration of healthy observers and the stimulus duration used in conventional perimetry, respectively.

### Procedures

**Fig. 3:**
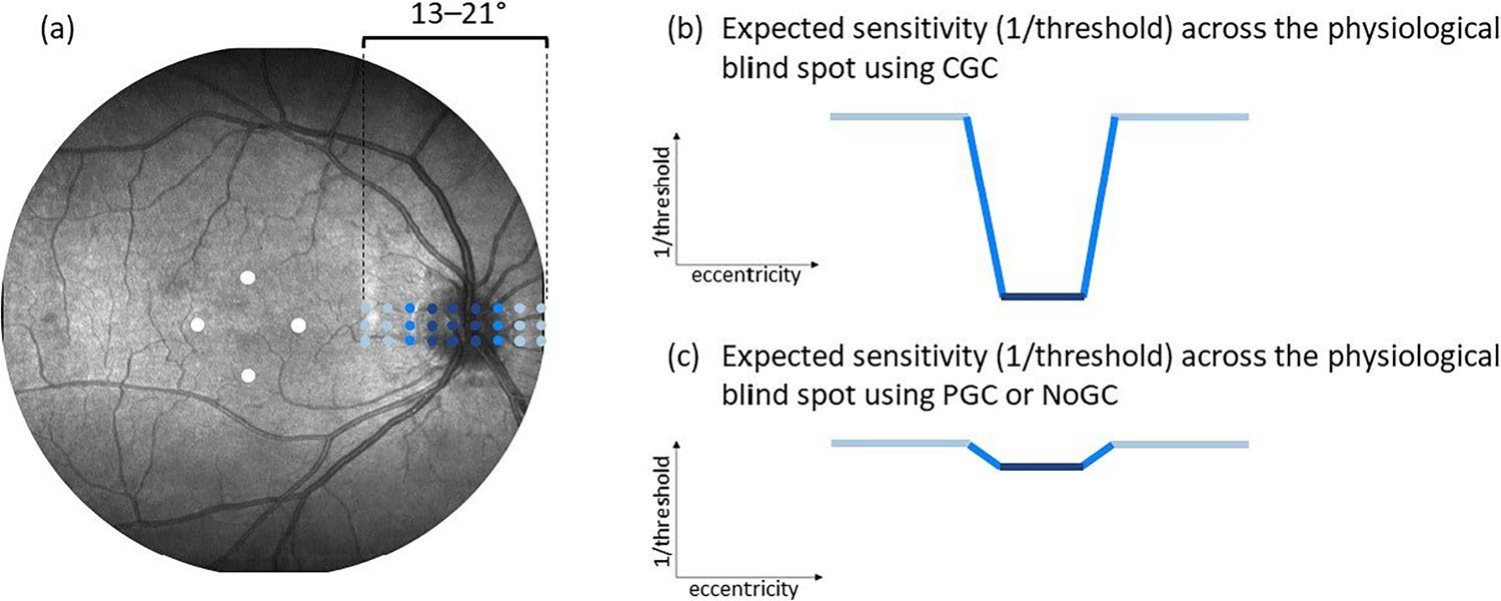
**(a)** Illustration of the visual field locations examined. Four stimuli were also shown within the central 3° of the visual field. **(b)** Schematic showing expected thresholds across the physiological blind spot using CGC in individuals with infantile nystagmus. **(c)** The expected partial or non-existent detection of the physiological blind spot using PGC and NoGC methods in individuals with infantile nystagmus

For the first part of this study, participants with infantile nystagmus and age-matched controls underwent visual field examinations using CGC and a stimulus grid centered over the physiological blind spot (optic nerve head). This provided a known area of visual field loss. NoGC and PGC visual field examinations were also carried out over the same area of the physiological blind spot. To account for any possible learning effect (Barkana et al., 2006; Liu et al., 2014; Marra & Flammer, 1991; Springer et al., 2005; Wild et al., 1999), these examinations were conducted at two separate visits and test order was randomized for all participants (median [IQR] time between visits: 8 days [7−13]). All visual field examinations were undertaken using a Goldmann III stimulus (0.43° diameter circle), with a 30 or 200 ms duration (separate examinations), 1.27 cdm^-2^ background luminance, 84.64 cdm^-2^ maximum stimulus luminance, and QUEST procedure (Yes/No paradigm; 𝛽 = 3.5; 𝛾 = 0; 𝛿 = 0.01) algorithm to estimate threshold (Watson & Pelli, 1983). Despite the ability of CGC and PGC to compensate for unstable fixation away from a central fixation target, participants were encouraged to fixate a central 2 × 2° fixation target (Thaler et al., 2013) throughout all examinations to maintain concentration and attention. A rest break was taken between each examination. Horizontal stimulus locations ranged from eccentricities of 13 to 21° (1° horizontal spacing between stimuli) and vertical locations ranged from eccentricities of – 1 to 1° above and below the fovea (1° vertical spacing between stimuli). Four stimuli were also presented in the central 3° of the visual field to maintain participant concentration and prevent habituation to the area of stimulus presentation. Figure 3 illustrates the visual field locations tested across the physiological blind spot. Since CGC aims to completely compensate for eye movements prior to and during stimulus presentation, we hypothesized that this method would more accurately detect the blind spot compared to PGC and NoGC methods. Figure 3 also shows a schematic to illustrate this hypothesis. In the case examples shown, thresholds (1/threshold [in cdm^‐2^]) from the second visit were plotted against all horizontal stimulus locations (13–21°) for the vertical location relative to the fovea that showed the greatest difference in threshold over the physiological blind spot (i.e., either – 1°, 0°, or 1°).

In the second part of this study focusing on healthy observers, 33 participants performed visual field examinations with CGC and PGC methods, and 15 of these individuals also carried out visual field examinations with the NoGC method. All participants were experienced in performing visual field examinations. To account for any learning effect and to measure test–retest variability, these examinations were repeated at two separate further visits and test order was randomized for all participants (median [IQR] time between visits: 7 days [4−11]). Examinations were completed using a 10° stimulus grid (68 locations, with a maximum eccentricity of 10° and a separation of 2°; similar to the 10–2 stimulus grid pattern of the HFA). It should be noted that all current commercially available microperimeters and perimeters are capable of presenting a 10° stimulus grid, but most commercial microperimeters are not capable of examining beyond 20° eccentricity (with the exception of one recent microperimeter, the Compass). Stimulus parameters and luminances were the same as described for the infantile nystagmus case examples. All visual field examinations included in this analysis had false-positive and false-negative rates below 15% (Heijl et al., 2012).

For both parts of this study, the eye with the better visual acuity was chosen as the test eye or, if both eyes had equal visual acuity, the right eye was tested by default. The fellow eye was patched. All examinations were performed with participants wearing full refractive correction for a working distance of 33 cm. Each participant underwent at least 3 min of adaptation to the background luminance of the screen prior to assessment.

### Statistical analysis

Statistical analyses were performed using the open-source statistical environments ﻿R (version 4.2.1, R Core Team 2022) and JASP (version 0.16.0, JASP Team 2021). Data were tested for normality using the Shapiro–Wilk test. All visual field data were converted to a right eye format. Threshold values are presented in units of luminance increment as compared to the background (cdm^-2^).

### Stimulus positional error

Eye tracking data were available to the system at 2000 Hz and the latest available gaze sample was used at each monitor refresh (130 Hz – maximum monitor refresh rate and overall operational rate of the system). This refresh rate produces a gaze-contingent stimulus update delay of around 8 ms; considerably lower than the 40 ms delay that is inherent with a 25 Hz microperimetry system. Since no system can precisely stabilize a stimulus without some level of temporal delay, at any given moment during a stimulus presentation there will be a small discrepancy between the *intended* stimulus location and the *actual* stimulus location; here we refer to this deviation as the ‘stimulus positional error’. Since NoGC does not compensate at all for eye movements, this method is expected to produce the highest stimulus positional error.

In healthy observers, stimulus positional error was calculated every time a stimulus was presented, and averaged over all stimulus presentations to produce a single error value for each examination. For each participant, these errors were then averaged across all visits and both stimulus durations to provide a single error value for each gaze-contingency method. A repeated-measures ANOVA was performed to determine the variation in stimulus positional error between CGC, PGC, and NoGC (with a within-subject factor of gaze-contingency method used). Bayes factors (BF_01_; prior scaling parameter = 0.707) are given alongside frequentist statistics for reference, and indicate, as a ratio, the relative weight of evidence for a difference between conditions versus the evidence of no difference between conditions. Ratios less than 1 indicate the data are more compatible with a difference; ratios more than 1 indicate the data are more compatible with no difference (Jarosz & Wiley, 2014; Jeffreys, 1961). Standardized effect sizes and their confidence intervals were also calculated.

There is a further form of temporal delay internal to the EyeLink 1000 Plus eye tracker, known as the end-to-end sample delay. It refers to the time taken for gaze position to be recorded by the EyeLink camera and processed by the EyeLink system, before being relayed back via an ethernet connection to the display system. However, for a 2000 Hz sampling rate, this delay is very small, i.e., approximately 1 ms (SR Research, 2019). This is unlikely to have any substantial effect on the accuracy of gaze-contingent stimuli in addition to the delay caused by monitor refresh rate.

### Accuracy and precision of visual field sensitivity estimates in healthy observers

To test the hypothesis that the test–retest variability of CGC is comparable to PGC and NoGC methods (using either a 30 or 200 ms stimulus duration) in healthy observers, test–retest intervals were established as the 5th and 95th percentiles of follow-up thresholds, as a function of baseline threshold (Artes et al., 2005; Wild et al., 1999). The step-by-step process describing how test–retest intervals were determined across all healthy observers can be found in ‘*Supplementary materials*’.

The presence or absence of a learning effect was also established across the three visits for each gaze-contingency method and stimulus duration. Further detail on the statistical analysis for this measure can also be found in ‘*Supplementary materials*’.

## Results

### Visual field examination in infantile nystagmus - Case examples

**Fig. 4:**
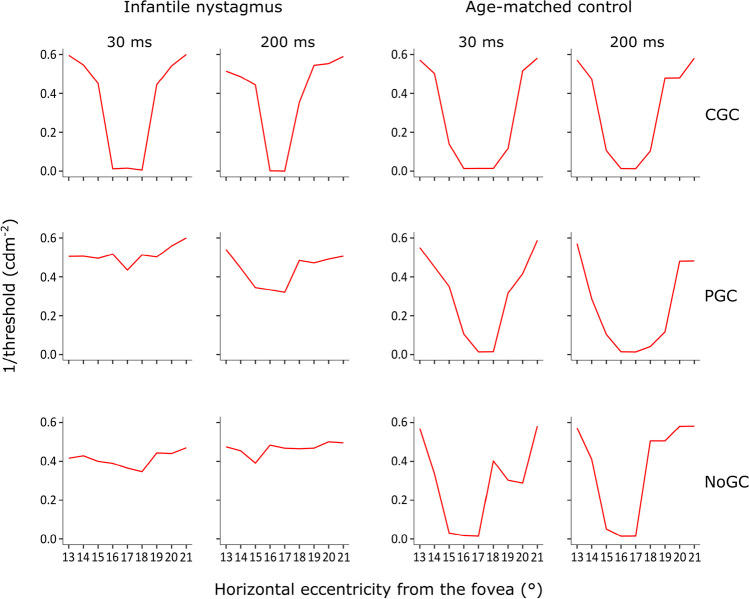
Case example I displaying threshold (1/cdm^-2^) as a function of horizontal stimulus eccentricity for visual field examinations conducted with each gaze-contingency method, at each stimulus duration, for a participant with infantile nystagmus (male, 34 years old) and an age-matched control (male, 33 years old)

**Fig. 5:**
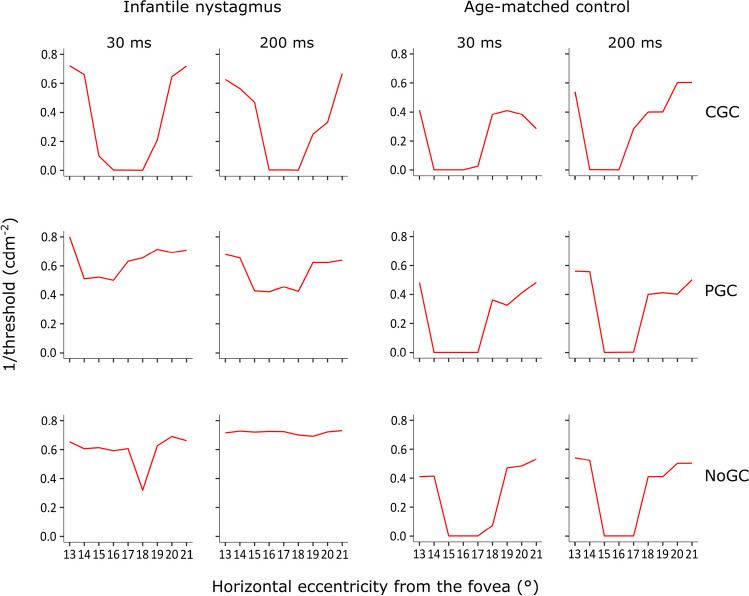
Case example II illustrating threshold (1/cdm^-2^) as a function of horizontal stimulus eccentricity for visual field examinations conducted with each gaze-contingency method, at each stimulus duration, for a participant with infantile nystagmus (female, 22 years old) and an age-matched control (female, 25 years old)

**Fig. 6:**
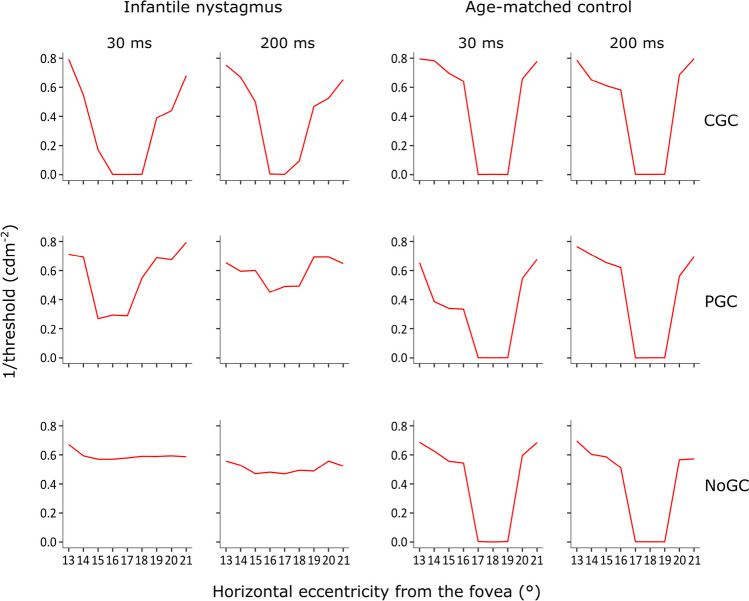
Case example III illustrating threshold (1/cdm^-2^) as a function of horizontal stimulus eccentricity for visual field examinations conducted with each gaze-contingency method, at each stimulus duration, for a participant with infantile nystagmus (male, 61 years old) and an age-matched control (male, 60 years old)

Figures 4, 5 and 6 illustrate examples of absolute scotomas detected in three cases of infantile nystagmus. In each figure, measured threshold (red line) across the area of the physiological blind spot is shown for examinations conducted with CGC (top panels), PGC (middle panels), and NoGC (bottom panels), with both a 30 ms and 200 ms stimulus duration. The data shown in each plot represent the vertical location relative to the fovea that showed the greatest decline in threshold over the physiological blind spot (i.e., either – 1°, 0°, or 1°). Successful detection of the physiological blind spot is shown by the decline in 1/threshold over the anatomical area of the optic nerve head (eccentricity from the fovea in degrees; *x-*axis). In all participants with infantile nystagmus, visual field examinations conducted using CGC were able to detect the physiological blind spot with better accuracy and consistency than PGC or NoGC. In age-matched controls, CGC, PGC, and NoGC were able to detect the physiological blind spot with equal accuracy.

### Stimulus positional error

**Fig. 7:**
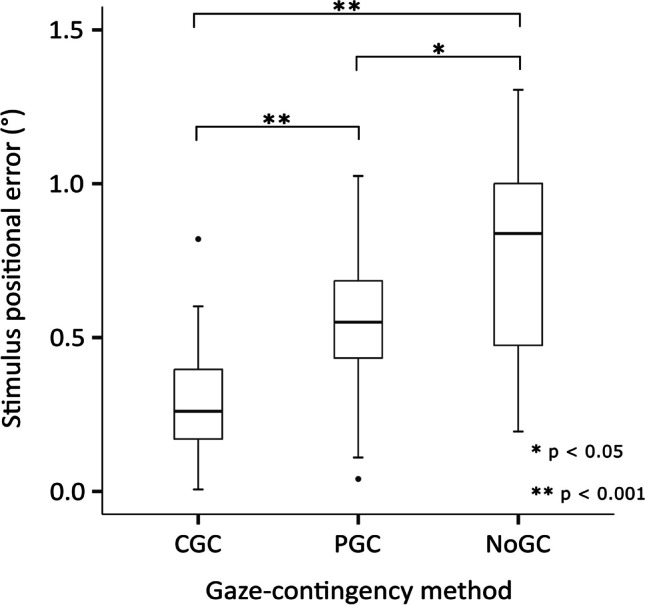
Stimulus positional error (in degrees) averaged across all stimulus presentations, and subsequently across all participants, all visits, and both stimulus durations. Box plot limits represent the maximum (*upper whisker*), upper quartile (*top of box*), median (*horizontal line in box*), lower quartile (*bottom of box*), and minimum (*lower whisker*) stimulus positional error

Figure 7 shows stimulus positional error (in degrees) for each gaze-contingency method conducted in healthy observers. A repeated-measures ANOVA showed a significant difference between these errors (F_1.68, 53.61_ = 32.32, η_p_^2^ = .50, 95% CI [0.30, 0.63], *p* < 0.001; BF_01_ < 0.001). Significant differences between pairwise combinations of gaze-contingency methods were also identified by post hoc comparisons (Bonferroni CGC/PGC *p* < 0.001; CGC/NoGC *p* < 0.001; PGC/NoGC *p* = 0.01; Bayesian post hoc BF_01_ CGC/PGC < 0.001; CGC/NoGC < 0.001; PGC/NoGC = 0.02). NoGC had the highest levels of stimulus positional error (mean stimulus positional error: CGC = ± 0.29°, PGC = ± 0.54°, NoGC = ± 0.81°).

Data showing the accuracy of visual field sensitivity estimates, test–retest variability, and the existence of any learning effects in healthy observers can be found in ‘*Supplementary materials*’.

## Discussion

This study introduces high-speed CGC perimetry, in which stimulus position is continuously updated at every monitor refresh (via eye tracking at 2000 Hz and an overall operational rate of 130 Hz using our hardware). We have demonstrated in preliminary data that CGC is better at detecting an absolute scotoma in individuals with infantile nystagmus compared to PGC and NoGC, using either a 30 or 200 ms stimulus duration. In all cases, PGC was able to detect the blind spot only to a certain extent in infantile nystagmus, and this occasionally varied with the stimulus duration used (mean depth of scotoma [1/cdm^-2^]: CGC/30 ms = 0.70 cdm^-2^, CGC/200 ms = 0.67 cdm^-2^ PGC/30 ms = 0.30 cdm^-2^, PGC/200 ms = 0.24 cdm^-2^, NoGC/30 ms = 0.19 cdm^-2^, NoGC/200 ms = 0.08 cdm^-2^). NoGC was poor at detecting the blind spot in all cases, presumably because there was no compensation for the continuous eye movements. As expected, all three gaze-contingency methods detected the blind spot more or less equally well in the age-matched controls, as these participants had stable fixation. These preliminary findings show that a more reliable assessment of visual field loss may be achieved by using CGC in patients with infantile nystagmus. Importantly, this result signifies that changes in stimulus sampling and retinal ganglion cell density are reduced with CGC in specified areas of the visual field, leading to a more precise estimate of visual field sensitivity in infantile nystagmus. This has crucial implications for relating visual field sensitivity to retinal changes in infantile nystagmus as well as other causes of unstable fixation, and is particularly relevant when studying highly localized structure–function relationships during the diagnosis of disease. Previous studies have used a combination of structural and functional data in patients without infantile nystagmus to provide enhanced estimates of the likelihood of changes in the condition over time (Medeiros et al., 2011; Russell et al., 2012; Zhu et al., 2014). Future studies will need to further investigate the ability of CGC to accurately characterize the relationship between visual field sensitivity and retinal structure in other areas of the visual field in infantile nystagmus. It is also notable that visual field stimulus processing in the visual pathway is a multi-stage process, and although CGC may help to address the problem of sampling at the level of the retinal ganglion cells in infantile nystagmus, it does not address how the solution applies to sampling and processing at the level of the visual cortex (high level spatial filters) (Swanson et al., 2004).

In healthy observers, NoGC had the largest stimulus positional error (± 0.81°). This was expected, as NoGC does not compensate for eye movements at all. PGC produced a significantly larger stimulus positional error compared to CGC, which was likely due to the continual update of stimulus position *during* a stimulus presentation with CGC, compared to updating the stimulus position prior to, but not during, stimulus presentation with PGC. This important finding highlights the advantage of CGC for highly precise gaze-contingent stimulus presentation, even in healthy observers with stable fixation. Furthermore, the stimulus positional error for CGC (± 0.29°) did not exceed the diameter of the stimulus (0.43°), indicating that for any given stimulus location, CGC was the only method to continually stimulate the same retinal locus throughout a visual field examination. As a secondary aim, we have also shown that CGC perimetry has equivalent accuracy and test–retest variability to PGC and NoGC methods in healthy observers (see ‘*Supplementary materials*’). It is important to note that the PGC and NoGC methods employed in this study are applications of existing paradigms present in commercial microperimeters and SAP, respectively. This is a strength of our study, as comparing CGC, PGC, and NoGC using the same eye tracking sampling rate, threshold estimating strategy, display parameters, and experimental platform, avoids complications that would have arisen had we attempted to compare CGC to existing commercial instruments.

A limitation of this study was that eye tracking was carried out by means of pupil/corneal reflex tracking (using the EyeLink 1000 Plus eye tracker). The disadvantage of this type of tracking is the inability to visualize the exact area of retinal stimulation throughout a visual field examination, in contrast to the live fundus tracking feature of microperimeters. However, pupil/corneal reflex tracking allows for much higher sampling rate, precision, and spatial resolution compared to current commercial microperimeters, which is of paramount importance for accurate gaze-contingency (Andersson et al., 2010). One limitation of our preliminary work in infantile nystagmus was that only a physiological *absolute* scotoma was assessed, which represents deep visual field loss. Even though this is an important first step in understanding the utility of CGC for detecting visual field loss in infantile nystagmus, glaucomatous visual field defects are often *relative* scotomas with varying depths of visual field loss, particularly in the early stages of the disease. Future work should investigate the detection of absolute scotomas in infantile nystagmus with a larger sample size, and also examine whether this method can detect relative scotomas related to retinal changes in individuals with infantile nystagmus and glaucoma.

This study demonstrates the ability of CGC to better detect an absolute scotoma in individuals with infantile nystagmus, compared to PGC and NoGC. This is the first published use of this method of gaze-contingent stimulus presentation in perimetry at a high monitor refresh rate and eye tracking speed. These results are an important precursor to establishing the potential of visual field examinations conducted with CGC in patients with unstable fixation, especially when relating visual field sensitivity to retinal changes during the diagnosis of disease. The ability of this method to stimulate precise retinal locations is also shown, as well as an equivalent accuracy and variability of CGC visual field sensitivity estimates compared to PGC and NoGC estimates in healthy observers. CGC has promising applications in psychophysical studies requiring precise retinal placement of visual stimuli, as well as in the development of perimetric techniques that are robust to moderate or high fixation instability.

### Supplementary Information

ESM 1 (DOCX 5027 kb)

## Data Availability

See ‘Open practices statement’.

## Code availability

See ‘Open practices statement’.
