# Supplementary material for: Reliability of gaze-contingent perimetry
Source: Behav Res Methods. 2023 Sep 11;56(5):4883–92. doi: 10.3758/s13428-023-02225-y (PMC11289009; doi:10.3758/s13428-023-02225-y)
Supplement: Supplementary file 1 — (DOCX 5027 kb) [file 13428_2023_2225_MOESM1_ESM.docx]

# Supplementary materials

## Statistical analysis

### Learning effects

To examine whether a learning effect was present between the three visits conducted with each gaze-contingency method and stimulus duration, a repeated-measures ANOVA of mean threshold was performed (within subject factors of first, second, or third visit). Bayes Factors (BF_01_; prior scaling parameter = 0.707) are given alongside frequentist statistics for reference. Standardized effect sizes and their confidence intervals are also displayed.

### Test-retest variability

Test-retest intervals were determined as the 5^th^ and 95^th^ percentiles of follow-up thresholds, relative to the baseline threshold (Artes et al., 2005; Wild et al., 1999). The following step-by-step process describes how test-retest intervals were determined across all participants (an example is also provided in Supplementary Figure 1) :

1. For a visual field examination conducted at Visit 1, baseline thresholds of a given value were extracted from each stimulus location resulting in that threshold value. In the example in Supplementary Figure 1, the threshold value extracted was 0.21 cdm^-2^.
2. Threshold values for each of the same stimulus locations identified in Step 1 were extracted from Visit 2 and Visit 3 visual field examinations.
3. The same process was then applied to each of the remaining participants whose baseline outcomes included locations with a threshold of 0.21 cdm^-2^. Following this, 5^th^ and 95^th^ percentiles were calculated from the range of thresholds obtained from all Visit 2 and Visit 3 stimulus locations identified in Step 2, for those locations with a baseline threshold of 0.21 cdm^-2^.

Steps 1-3 were repeated for all other thresholds obtained across all participants. Although thresholds in this study were measured to the nearest 0.001 cdm^-2^, thresholds used to calculate these test-retest intervals were quantized to the nearest 0.01 cdm^-2^. This enabled a large proportion of participants to be captured for a given threshold value. Furthermore, since there was no learning effect present between the three visits (repeated-measures ANOVA of mean threshold, all p > 0.05 for each gaze‑contingency method and stimulus duration; see Supplementary Table 1), the order of the tests was regarded as interchangeable, meaning that Visit 2 and Visit 3 thresholds were also used as baseline thresholds. This enabled approximately 400-500 threshold values to be utilized as baseline thresholds for each gaze-contingency method and stimulus duration tested.


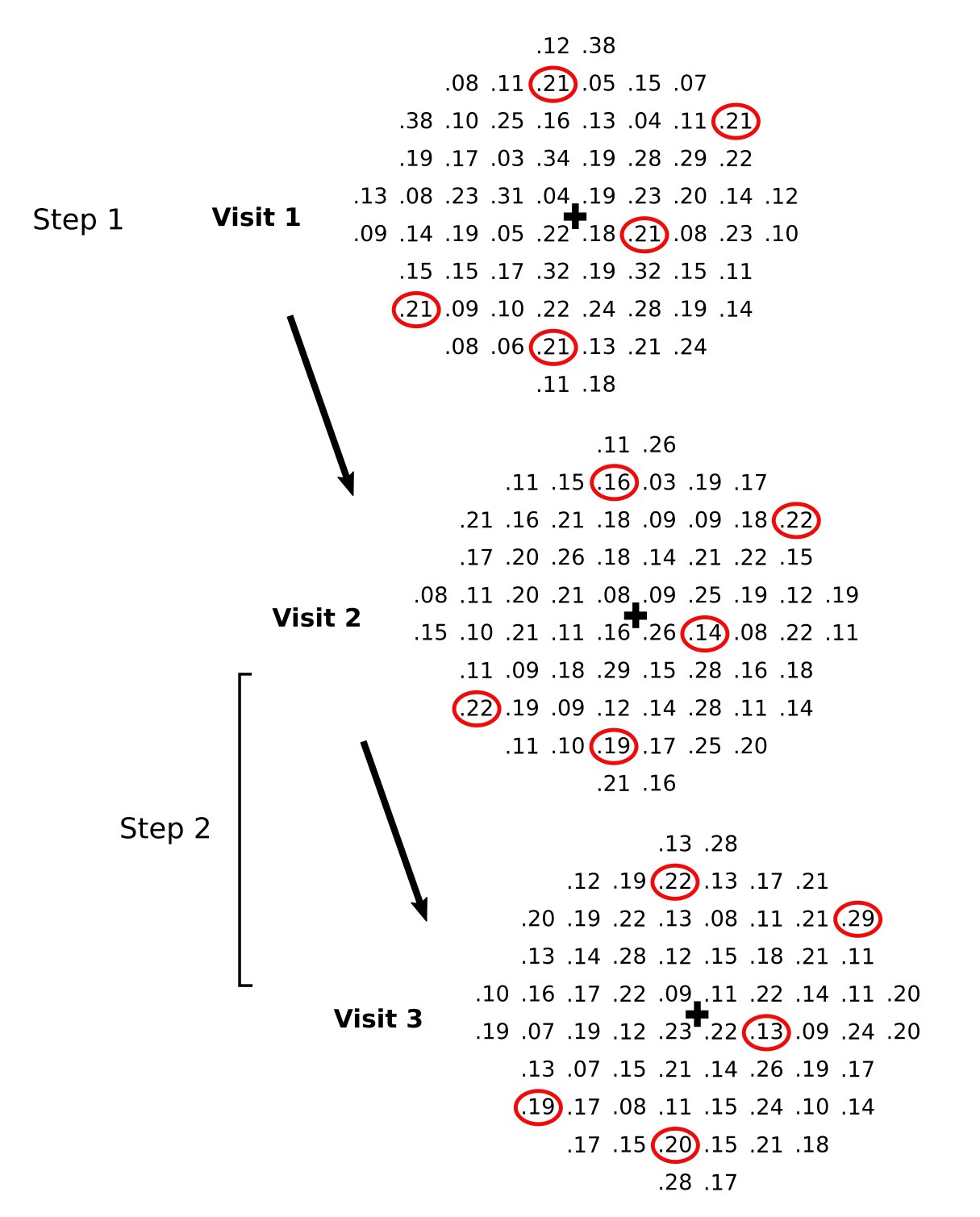


Supplementary Figure 1. Analysis steps to determine test-retest intervals for an example participant. In Step 1, baseline thresholds of 0.21 cdm^-2^ were extracted from each stimulus location resulting in that threshold value. In Step 2, threshold values from Visit 2 and Visit 3 are extracted from each of the same stimulus locations identified in Visit 1. In Step 3 (not shown in this figure), the same process was applied to all other participants whose baseline outcomes included locations with a threshold of 0.21 cdm^-2^. Finally, 5^th^ and 95^th^ percentile values were calculated from the range of follow-up thresholds identified in Step 2 across all participants, for a given baseline threshold of 0.21 cdm^-2^.

To examine the variance between gaze-contingency methods and stimulus durations, Bland‑Altman plots were derived for comparisons of mean threshold (across all stimulus locations for each participant) and mean threshold at each stimulus location (‘pointwise’; across all participants). Comparisons were made between the six different combinations of gaze-contingency methods and stimulus durations, and bias and 95% limits of agreement were calculated for each comparison (Bland & Altman, 1986).

## Results

Mean thresholds (averaged across all stimulus locations) were not significantly different from a normal distribution for each gaze-contingency method tested with either a 30 or 200 ms stimulus duration (Shapiro-Wilk test; all p > 0.05).

### Accuracy of visual field sensitivity estimates

Supplementary Figure 2 shows box plots of thresholds obtained with each combination of gaze-contingency method and stimulus duration across all participants. For each stimulus duration, threshold values were similar across all gaze-contingency methods. However, for all gaze-contingency methods, thresholds obtained using a 30 ms stimulus duration were higher than those obtained using a 200 ms duration.


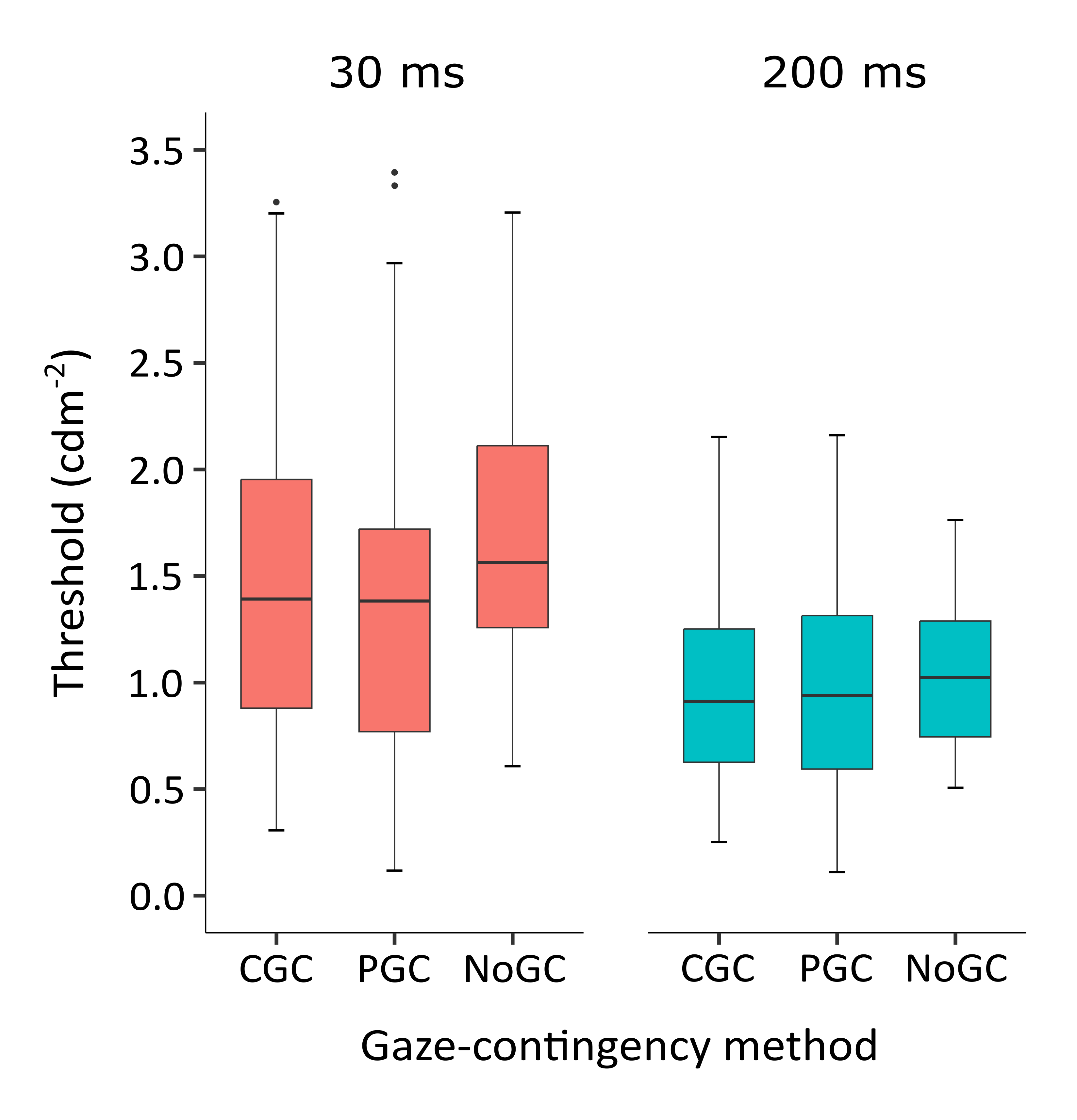


Supplementary Figure 2. Box plots showing thresholds obtained with each gaze-contingency method using both stimulus durations. Box plot limits represent the maximum (upper whisker), upper quartile (top of box), median (horizontal line in box), lower quartile (bottom of box), and minimum (lower whisker) values. Outliers are represented by black dots.

### Learning effects

A repeated-measures ANOVA of mean threshold did not suggest the presence of a significant learning effect across all three visits for any gaze-contingency method tested using either stimulus duration (Supplementary Table 1).

Supplementary Table 1. Repeated-measures ANOVA (with Bayes factors) to assess significant differences in mean thresholds across all three visits (within subject factor of visit), for each combination of gaze-contingency method and stimulus duration.

| **Gaze-contingency method** | **Stimulus duration (ms)** | **Repeated-measures ANOVA** | **Bayes Factor (BF_01_)** |
| --- | --- | --- | --- |
| CGC | 30 | F_2,32_= 2.27, η_p_^2^ = 0.07, 95% CI [0.001, 0.31], p = 0.12 | 1.84 |
|  | 200 | F_2,32_= 0.93, η_p_^2^ = 0.03, 95% CI [0.001, 0.21], p = 0.40 | 5.23 |
| PGC | 30 | F_2,32_= 1.14, η_p_^2^ = 0.03, 95% CI [0.001, 0.23], p = 0.33 | 4.23 |
|  | 200 | F_2,32_= 2.91, η_p_^2^ = 0.08, 95% CI [0.002, 0.34], p = 0.07 | 1.11 |
| NoGC | 30 | F_2,14_= 1.05, η_p_^2^ = 0.07, 95% CI [0.001, 0.23], p = 0.37 | 2.99 |
|  | 200 | F_2,14_= 1.54, η_p_^2^ = 0.10, 95% CI [0.003, 0.26], p = 0.25 | 2.19 |

### Test-retest variability

To demonstrate the variability of thresholds obtained with each gaze-contingency method and stimulus duration, Supplementary Figure 3 shows test-retest intervals for each gaze-contingency method and stimulus duration, across all participants. These are displayed as the 5^th^ (purple dots) and 95^th^ (green dots) percentiles of follow-up thresholds plotted against baseline threshold. Curves were fitted through the percentile data points (black lines). For each baseline threshold measured, test-retest intervals (shaded regions) describe the range within which 90% of follow-up thresholds are likely to fall. For all gaze-contingency methods, 90% test-retest intervals were larger and the percentile data spanned a larger range of thresholds when using 30 ms stimulus durations compared to 200 ms stimulus durations. However, for each stimulus duration, 90% test-retest intervals were relatively similar across the three gaze-contingency methods.


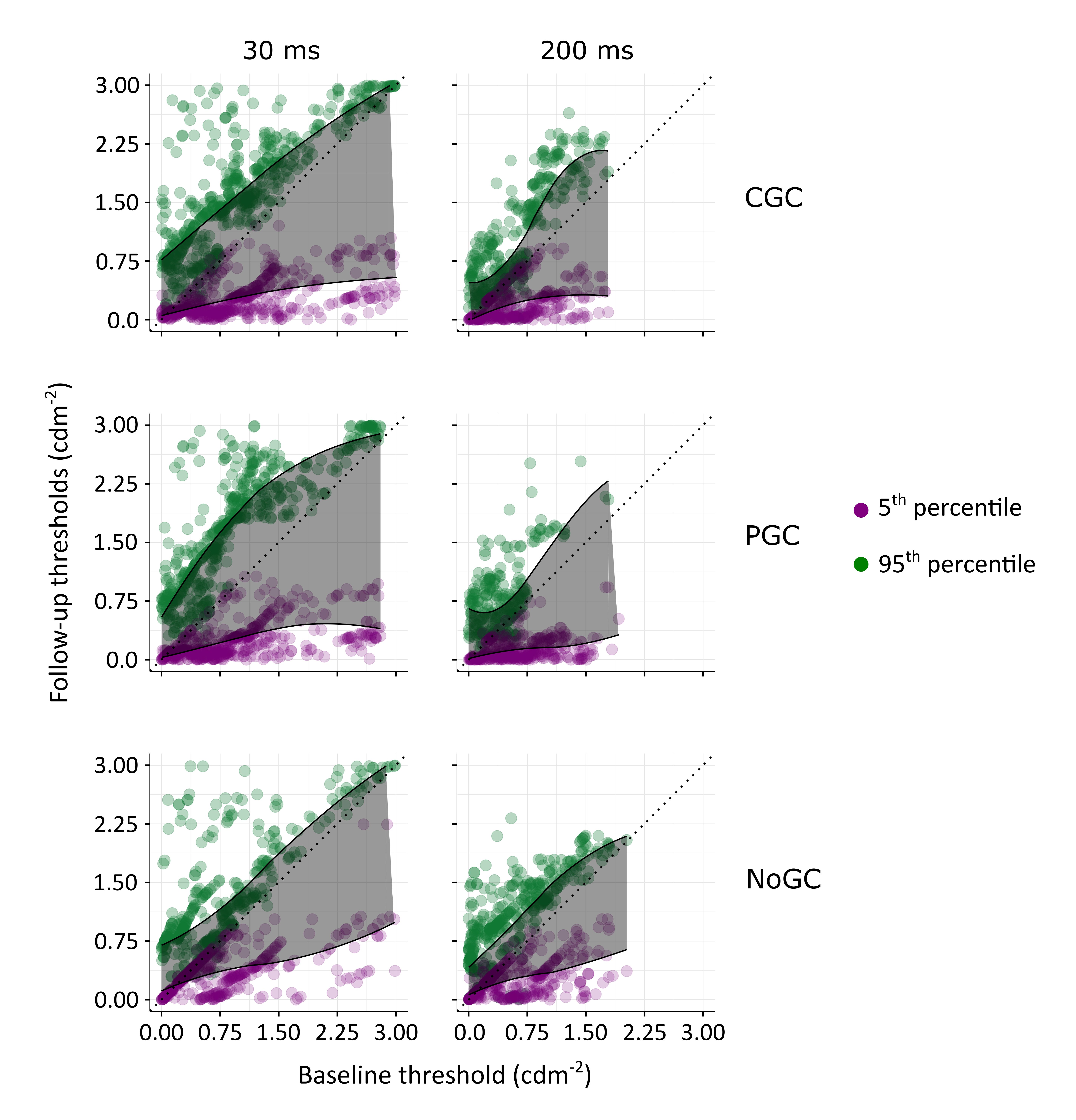


Supplementary Figure 3. Test-retest intervals displayed as the 5^th^ and 95^th^ percentiles of follow-up thresholds as a function of the mean threshold at baseline across all participants and stimulus locations for each gaze-contingency method and stimulus duration. Smooth curves were fitted using a locally weighted regression (weighted least squares; span = 1.0) (solid black lines). Shaded areas indicate the 90% test-retest intervals over the range of follow-up thresholds obtained. Dashed diagonal line: line of unity.

Bland-Altman plots in Supplementary Figure 4 show mean threshold comparisons between gaze-contingency methods. For each comparison, the variance of mean thresholds was greater for stimuli presented at 30 ms than those presented at 200 ms, although similar variance was found between the gaze-contingency methods for a given stimulus duration. Furthermore, there was no clear relationship between the magnitude of threshold and the variance between gaze-contingency methods with either stimulus duration, i.e., as mean threshold increased, the difference between methods did not increase or decrease.


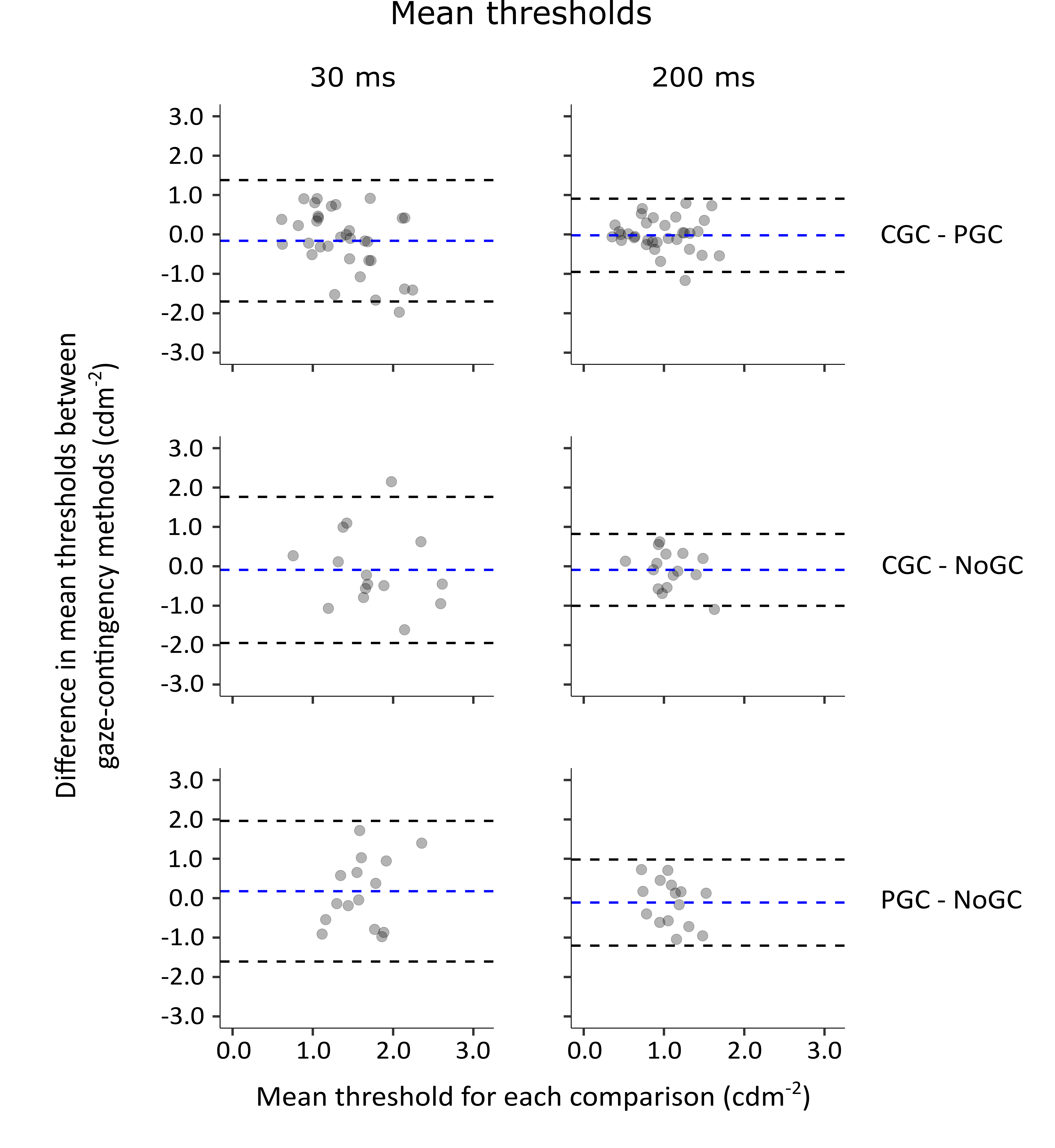


Supplementary Figure 4. Bland-Altman plots to represent the agreement of mean thresholds across all stimulus locations and all visits for each participant (each point represents a participant) between different gaze-contingency methods for stimulus durations of 30 ms and 200 ms. The differences plotted show CGC mean thresholds subtracted from PGC mean thresholds, and CGC or PGC mean thresholds subtracted from NoGC mean thresholds. The horizontal blue dotted line represents the mean difference and the black dotted lines represent the 95% limits of agreement.

Bland-Altman plots in Supplementary Figure 5 show comparisons of mean threshold at each stimulus location (‘pointwise’) between gaze-contingency methods. For each comparison, the variance of mean pointwise thresholds was similar for each stimulus duration, but differed between the two durations (although this difference was smaller than the difference between stimulus durations in Supplementary Figure 4). For comparisons between CGC and NoGC, and between PGC and NoGC, there was a shift of the mean difference between methods (horizontal dashed blue line) away from zero (i.e., towards 1.0).


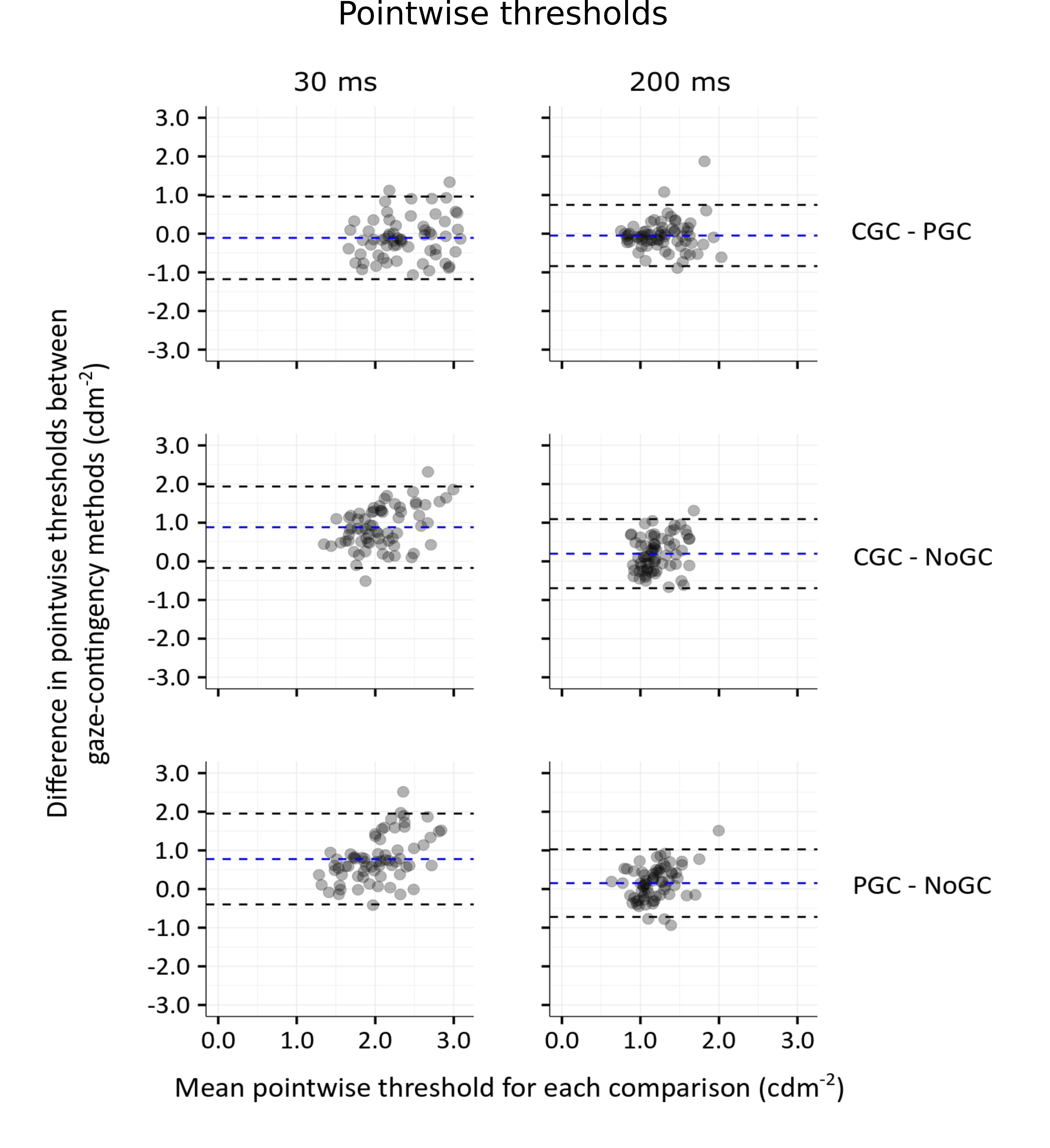


Supplementary Figure 5. Bland-Altman plots showing the agreement of mean pointwise thresholds across all visits and all participants for each stimulus location (each point represents a stimulus location) between different gaze-contingency methods for stimulus durations of 30 ms and 200 ms. The differences plotted show CGC pointwise thresholds subtracted from PGC pointwise thresholds, and PGC or CGC pointwise thresholds subtracted from NoGC pointwise thresholds. The horizontal blue dotted line represents the mean difference and the black dotted lines represent the 95% limits of agreement.

## Supplementary Discussion

Threshold values and test-retest intervals were similar across CGC, PGC, and NoGC when examined separately with each stimulus duration, although both thresholds and intervals were larger for 30 ms durations. Also, the difference between methods was larger for stimulus durations of 30 ms (for both mean and pointwise threshold comparisons; see Bland Altman plots in Supplementary Figure 4 and 5). This may be in part due to the differences between step sizes in terms of energy (increment luminance × duration × area) for a 30 ms and 200 ms stimulus, when presented at different intensities. With the area of stimuli fixed in this study (Goldmann III; 0.43° diameter), step size was dependent on stimulus duration (either 30 ms or 200 ms) and changes in luminance (cdm^-2^). As a result, energy step sizes between stimuli of common intensities are different for 30 ms and 200 ms stimulus durations. For example, for a 200 ms stimulus, the step size in energy terms for a 0.1 cdm^‑2^ increment change in luminance is 0.003 cdm^-2^·s·deg^2^ (0.1 cdm^‑2^ × 0.2 s × 0.15 deg^2^). However, for a 30 ms stimulus, the step size in energy terms for the same 0.1 cdm^-2^ increment change in luminance is 0.0005 cdm^-2^·s·deg^2^ (0.1 cdm^‑2^ × 0.03 s × 0.15 deg^2^). This means that, for the same increment change in luminance, the energy step size is 6× larger for a 200 ms stimulus duration compared to a 30 ms stimulus duration. This is particularly important near threshold, where larger step sizes will likely result in more repeatable thresholds.

There were no significant learning effects across all three visits with either CGC, PGC, or NoGC. However, intersession learning effects have been demonstrated many times with SAP in healthy observers (Castro et al., 2008; Heijl et al., 1989; Yenice & Temel, 2005) and in patients with glaucoma (Heijl & Bengtsson, 1996; Wild et al., 1989, 1991). Learning effects are typically most pronounced between the first two examinations (Castro et al., 2008; Flammer et al., 1984; Wild et al., 1991), and can also be influenced by the previous perimetric experience of the observer (Heijl et al., 1989). The absence of learning effects in the present study was likely due to the previous perimetric experience of our participants.

## References

Artes, P. H., Hutchison, D. M., Nicolela, M. T., Leblanc, R. P., & Chauhan, B. C. (2005). Threshold and variability properties of matrix frequency-doubling technology and standard automated perimetry in glaucoma. *Investigative Ophthalmology & Visual Science*, *46*(7), 2451–2457. https://doi.org/10.1167/iovs.05-0135

Bland, J. M., & Altman, D. G. (1986). Statistical methods for assessing agreement between two methods of clinical measurement. *The Lancet*, *327*(8476), 307–310.

Castro, D. P. E., Kawase, J., & Melo Jr., L. A. S. (2008). Learning effect of standard automated perimetry in healthy individuals. *Arquivos Brasileiros de Oftalmologia*, *71*(4), 523–528. https://doi.org/10.1590/s0004-27492008000400011

Flammer, J., Drance, S. M., & Zulauf, M. (1984). Differential light threshold: short-and long-term fluctuation in patients with glaucoma, normal controls, and patients with suspected glaucoma. *Archives of Ophthalmology*, *5*(102), 24–26.

Heijl, A., & Bengtsson, B. (1996). The effect of perimetric experience in patients with glaucoma. *Archives of Ophthalmology*, *1*(114), 19–22.

Heijl, A., Lindgren, G., & Olsson, J. (1989). The effect of perimetric experience in normal subjects. *Archives of Ophthalmology*, *107*(1), 81–86. https://doi.org/10.1001/archopht.1989.01070010083032

Wild, J. M., Dengler-Harles, M., Searle, A. E. T., O’Neill, E. C., & Crews, S. J. (1989). The influence of the learning effect on automated perimetry in patients with suspected glaucoma. *Acta Ophthalmologica*, *67*(5), 537–545.

Wild, J. M., Pacey, I. E., O’Neill, E. C., & Cunliffe, I. A. (1999). The SITA perimetric threshold algorithms in glaucoma. *Investigative Ophthalmology & Visual Science*, *40*(9), 1998–2009.

Wild, J. M., Searle, A. E. T., Dengler-Harles, M., & O’Neill, E. C. (1991). Long-term follow-up of baseline learning and fatigue effects in the automated perimetry of glaucoma and ocular hypertensive patients. *Acta Ophthalmologica*, *69*(2), 210–216. https://doi.org/10.1111/j.1755-3768.1991.tb02713.x

Yenice, O., & Temel, A. (2005). Evaluation of two Humphrey perimetry programs: full threshold and SITA standard testing strategy for learning effect. *European Journal of Ophthalmology*, *15*(2), 209–212. https://doi.org/10.1177/112067210501500205
